# Significance of Volatile Organic Compounds to Secondary Pollution Formation and Health Risks Observed during a Summer Campaign in an Industrial Urban Area

**DOI:** 10.3390/toxics12010034

**Published:** 2024-01-01

**Authors:** Li Cao, Qihui Men, Zihao Zhang, Hao Yue, Shijie Cui, Xiangpeng Huang, Yunjiang Zhang, Junfeng Wang, Mindong Chen, Haiwei Li

**Affiliations:** 1 Jiangsu Key Laboratory of Atmospheric Environment Monitoring and Pollution Control, Collaborative Innovation Center of Atmospheric Environment and Equipment Technology, School of Environmental Science and Engineering, Nanjing University of Information Science and Technology, Nanjing 210044, China; 2 Shanghai Key Laboratory of Atmospheric Particle Pollution and Prevention, Department of Environmental Science and Engineering, Fudan University, Shanghai 200438, China; 3 State Environmental Protection Key Laboratory of Formation and Prevention of Urban Air Pollution Complex, Shanghai Academy of Environment Sciences, Shanghai 200233, China

**Keywords:** VOCs, chemical characteristics, secondary pollution, source apportionment, health risks, industrial urban areas

## Abstract

The chemical complexity and toxicity of volatile organic compounds (VOCs) are primarily encountered through intensive anthropogenic emissions in suburban areas. Here, pollution characteristics, impacts on secondary pollution formation, and health risks were investigated through continuous in-field measurements from 1–30 June 2020 in suburban Nanjing, adjacent to national petrochemical industrial parks in China. On average, the total VOCs concentration was 34.47 ± 16.08 ppb, which was comprised mostly by alkanes (41.8%) and halogenated hydrocarbons (29.4%). In contrast, aromatics (17.4%) dominated the ozone formation potential (OFP) and secondary organic aerosol formation potential (SOAFP) with 59.6% and 58.3%, respectively. Approximately 63.5% of VOCs were emitted from the petrochemical industry and from solvent usage based on source apportionment results, followed by biogenic emissions of 22.3% and vehicle emissions of 14.2%. Of the observed 46 VOC species, hexachlorobutadiene, dibromoethane, butadiene, tetrachloroethane, and vinyl chloride contributed as high as 98.8% of total carcinogenic risk, a large fraction of which was ascribed to the high-level emissions during ozone pollution episodes and nighttime. Therefore, the mitigation of VOC emissions from petrochemical industries would be an effective way to reduce secondary pollution and potential health risks in conurbation areas.

## 1. Introduction

Volatile organic compounds (VOCs) are important precursors of tropospheric ozone (O_3_) and secondary fine particulate matter (PM_2.5_), which play a significant role in complex air pollution in conurbation areas in China, such as the Beijing-Tianjin-Hebei (BTH), the Yangtze River Delta (YRD), and the Pearl River Delta (PRD) [1,2]. O_3_ is a significant driver of photochemical smog [3,4,5]. Aside from global warming effects, the increasingly high ground-level O_3_ concentrations can induce adverse health risks for humans. Acute exposure to O_3_ can impair the respiratory system, leading to various symptoms such as breathing difficulties, airway inflammation, asthma, emphysema, and chronic bronchitis [6,7]. In addition, aboveground biological processes can be affected by O_3_ [8]. Leaf photosynthesis and associated antioxidant capacity are reduced with elevated O_3_ [9,10]. Moreover, crop growth is susceptible to O_3_ stress, which in turn affects food security [11,12]. High concentrations of VOCs and the associated VOCs-limited O_3_ formation regime have been well recognized as the key for synergistic control of both O_3_ and haze pollution [8,9,10]. Despite remarkable decreases in the emissions of primary pollutants (e.g., SO_2_, NO_x_, and particulate matter), the VOC pollution in urban environments still exhibits an increasing or non-decreasing trend due to complex sources, speciation, and chemical transformations of VOCs [13].

Nowadays, the increasing numbers in major cities beyond downtown areas are contributing to serious VOC pollution problems in China owing to decades of efforts to emigrate the non-essential industrial functions (e.g., coal-based power generation and petrochemical industries) to suburban or rural areas [14,15]. As such, industrial–vehicular sources have become the dominant emission sectors for VOCs in suburban areas, contributing to more than 28% of the total volatile organic compound (TVOC) mixing ratio observed in an urban–industrial complex metropolis [16,17]. Abundant C_8_ and C_9_ aromatics are attributed to the vehicular–industrial source, which could contribute to 23% of the total OH reactivity [16]. Past studies have shown that the average ratio of toluene to benzene was 5.7 ppb ppb^−1^, indicating primary contributions of toluene from industrial and vehicle emissions [16,18]. In addition, intensive VOC emissions from the petrochemical industry have dominated in the industrial urban area over a long term, and reduction of VOC emissions from the petrochemical industrial processes could be a promising way to alleviate O_3_ pollution [19]. Consequently, the complex air pollution linked to O_3_ and fine particulate matter has increased in industrial urban areas, driven by the chemically complicated air-pollution processes. Reduction in anthropogenic VOC emissions from industrial and traffic sources is still a priority in current air-pollution control for fast-developing cities [14]. However, the sources of VOCs are highly complex because their fast emission and fast oxidation make the source apportionment of ambient hydrocarbons more challenging in such urban–industrial complex areas [4].

Intensive industrial activities lead to a series of environmental problems. Besides precursors of secondary pollution, most VOCs exhibit detrimental effects and thus are classified as suspected carcinogens to humans [20,21,22,23,24]. More studies have shown that occupational health risks for workers are corelated with serious exposure to high-level VOCs from industrial activities. A large variety of industrial-related VOCs, such as alkenes, aldehydes, aromatics, and halohydrocarbons, are irritations of eyes, skin, respiratory mucosa, and nervous system; long-term exposure to these VOCs can increase the risk of neurasthenia, deformities, and cancers. Exposure to benzene homologues such as toluene, ethylbenzene, and xylene can cause acute neurological poisoning symptoms (headache, nausea, fatigue, and nervous and immune system injuries) [25,26,27]. Benzene and 1,3-butadiene have been identified as common toxins for both non-carcinogenic and carcinogenic risks of VOCs [28]. Some specific VOCs containing unpleasant odorous compounds like organic sulfides, amines, and chlorides pose additional risk factors to human health, which has increasingly become a currently urgent social issue in public complaints [27]. Reduction of VOCs from industrial sources could help in alleviating the olfactory nuisance and adverse health issues related to odor pollution for workers and residents. However, the quantification of the health risk of VOCs for surrounding residents has rarely been undertaken in industrial urban areas because it can be difficult to accurately estimate their exposure concentration, time, and frequency. These studies differ from risk assessment for workers, as those modeling parameters can be quantified (e.g., daily exposure time of 8 h day^−1^ [29]). Previous studies on health risk assessments of VOCs are almost completely focused on the odor pollution in municipal solid waste disposal facilities [30].

Studies of the pollution characteristics and health risk assessments of VOCs in chemical and industrial parks are scarce in the YRD region (one of the largest industrial bases in China). As the capital of Jiangsu Province, Nanjing is one of the representative mega industrial cities in the YRD region, including many industrial fields such as chemical, petrochemical, and steel. Herein, the concentration, chemical composition, secondary pollution feedback, source profiles, and associated health risks of ambient VOCs were fully investigated in an industrial urban area in Nanjing. In addition, this summertime campaign (1–30 June 2020) was organized into O_3_ episode days and non-O_3_ episode days for further comparisons, according to the maximum daily 8-h average O_3_ concentration (MDA8 O_3_) exceeding 160 μg m^−3^ as the Class II standard in China [31]. On the basis of source identification through the positive matrix factorization (PMF) model analysis, the source-specific health risks of VOCs were quantified and evaluated by estimating their hazard index (HI) and lifetime cancer risk (LCR). This work can provide some insights into formulating effective strategies for industrial–vehicular VOCs control and the elimination of dominating harmful or toxic components.

## 2. Materials and Methods

### 2.1. Sampling Site

**Figure 1:**
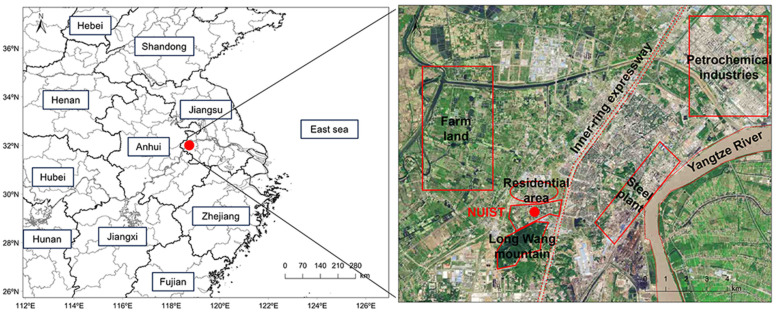
Location of the sampling site labeled with red dot and the distribution of different types of functional areas including industry, traffic, residential areas in its surroundings.

As shown in Figure 1, the sampling site is located at an integrated meteorological observation base (118.710977° E, 32.204100° N) on the campus of Nanjing University of Information Science and Technology (NUIST) in northern suburban Nanjing, the capital of Jiangsu Province, which is one of the largest industrial bases in the western YRD region of China. The sampling site is surrounded by industrial factories, traffic, and residential areas. The site is close to the inner expressway in the east (1 km away), with high traffic flows. The national petrochemical industrial parks are located approximately 10 km northeast of the sampling site. Additionally, several iron and steel plants and power plants are within 4 km of the site. Hence, this site represents a typical industrial urban area with enhanced industrial activities and prevalence of vehicle use.

### 2.2. On-Site Measurements

Ambient VOCs in the air were monitored in real time using an online single-photon ionization time-of-flight mass spectrometer (SPI-ToF-MS 3000, Guangzhou Hexin Instrument Co., Ltd., Guangzhou, China) from 1–30 June 2020, during which strong solar irradiation and high temperatures can incur O_3_ pollution over a typical year. This SPI-MS instrument is comprised of a polydimethylsiloxane (PDMS) membrane inlet system, vacuum ultraviolet (VUV) light irradiation system, single-photon ionization source, and mass spectrometry analysis system for data acquisition. The limits of detection (LOD) of toluene and xylene were kept to 0.16 ppbv for 1 s measurement with a scan frequency of 10 kHz. The standard gases included photochemical assessment monitoring stations (PAMS) and toxic organics−15 (TO15) (Guangzhou Hexin Instrument Co., Ltd., Guangzhou, China). Ultrapure nitrogen was used as the carrier gas via a portable gas dilution system (Sabio 2010, SABIO company, Round Rock, TX, USA). Standard gases were diluted to concentrations of 0, 1, 2, 5, 10, 20, and 40 ppb for a 7-point calibration. The correlation coefficient (*R*^2^) of the calibration curves was ~0.99 for speciated VOCs, and each calibration was maintained at least five times through standard gases until equilibrium was reached. The detailed VOC sampling and measurement through of SPI-ToF-MS are given in Supplementary Materials. In total, 46 compounds were measured, including 6 benzene homologues, 11 alkanes, 5 alkenes, 11 halohydrocarbons, and 13 other VOCs (5 nitrogen-containing compounds and 8 oxygenated compounds), as shown in Table S1. These speciated VOCs were selected as representative of industrial–vehicular-related anthropogenic emissions, and they also showed a high SPI signal sensitivity (*R*^2^ = 0.99) according to the calibration. Additional trace gases including O_3_, NO, NO_2_, CO, and SO_2_ and meteorological parameters including temperature, relative humidity, wind speed, and wind direction were measured at a one-minute interval at the air quality monitoring station.

### 2.3. Data Analysis

#### 2.3.1. Ozone Formation Potential

Maximum incremental reactivity (*MIR*) is used to calculate the ozone generation potential (*OFP*) of VOCs, which is based on the effect of input and output of unit species on ozone generation. Ozone formation is sensitive to changes in concentrations of VOCs [32,33]. This method can evaluate the relative contributions of dominant VOCs species to ozone formation along with the photochemical reaction pathways. The *OFP* is calculated by the following equation [34]:

(1) $$OFP=\frac{1}{M_{O_{3}}}\sum {MIR}_{i}\times {concentration}_{i}\times M_{i}$$

where $M_{O_{3}}$ is the relative molecular mass of ozone (g mol^−1^); ${MIR}_{i}$ is the *MIR* of compound_i_ ($g_{O_{3}}/g_{VOCs}$), which can be obtained from the previous literature [35]; *concentration_i_* is the volumetric concentration of ${compound}_{i}$ (ppb); $M_{i}$ represents the relative molecular mass of ${compound}_{i}$ (g mol^−1^).

#### 2.3.2. Secondary Organic Aerosol Formation Potential

*VOCs* have additional important effects on photochemistry in tropospheric atmosphere, which generates an abundance of *SOA* [36]. While quantifying secondary organic aerosol formation potential (*SOAFP*) is complicated, the fractional aerosol coefficient (*FAC*) is used to explore the contribution of each species to *SOA* formation [37]. *SOAFP* is thus calculated by the following Equations (2) and (3):

(2) $${SOAFP}_{i}={VOC}_{S_{i}}\times {FAC}_{i}$$

(3) $${VOC}_{T_{i}}={VOC}_{S_{i}}\times (1-F_{i})$$

where *SOAFP_i_* is the formation potential of secondary aerosols (µg m^−3^) of the species *i*; *VOCs_i_* is the initial concentration of species emitted from pollution sources (µg m^−3^); *FAC_i_* is the conversion coefficient of the species *i*; *VOC_Ti_* is the species concentration of *VOCs* (µg m^−3^) at a given time; and *F_i_* is the consumption coefficient of species in the atmosphere. *FAC_i_* and *F_i_* can be obtained from the literature [38].

#### 2.3.3. The PMF Model

The PMF receptor model is an effective multivariate factor analysis tool based on the observational results and thus has been widely applied to resolve potential sources of atmospheric pollutants [39,40]. The PMF 5.0 developed by the U.S. Environmental Protection Agency (USEPA) was used to identify dominant VOCs sources and evaluate their contributions to VOCs. PMF can categorize the observed data into two matrices, i.e., factor contributions and factor profiles, on the basis of concentration and uncertainty of each species [41]. The uncertainty (*unc*) of different species is calculated by the following equations:

(4) $$unc=\frac{5}{6}\times MDL\;(C\leq MDL)$$

(5) $$unc=\sqrt{{\left(Error\;Fraction\times c\right)}^{2}+{\left(0.5\times MDL\right)}^{2}}$$

where *MDL* represents methods detection limit; *C* is the concentration of species, and error fraction is determined by the performance of the instrument and activity of the species [42,43]. The selected species are divided into three categories according to signal-to-noise ratio (S/N) and the percent of missing value. A group of 26 major species of VOCs was input into the model analysis, which are representative traces for different sources with relatively high abundance and S/N value. These selected species can make large contributions to the total concentration of VOCs if the S/N is greater than 2. In addition, the ratio of Q(true)/Q(robust) was used to examine the modeling results. When the ratio is less than 1.5, the results are thought to be reliable and credible, while a ratio > 1.5 indicates that a large number of outliers in the model requires further data screening [44].

#### 2.3.4. Health Risk Assessment

Hazard index (*HI*) and lifetime cancer risk (*LCR*) were estimated to assess residents’ noncarcinogenic and carcinogenic risks via inhalation exposure to VOCs according to the following equations [27,45,46]:

(6) $$HI=\frac{\left(C_{i}\times ET\times EF\times ED\right)}{365\times {AT}_{nca}\times 24}\times \frac{1}{{RfC}_{i}}$$

(7) $$LCR=\frac{\left(C_{i}\times ET\times EF\times ED\right)}{365\times {AT}_{ca}\times 24}\times {IUR}_{i}$$

where *C_i_* (μg m^−3^) is the daily ambient concentrations of VOCs. According to the Chinese Exposure Factors Handbook (Adults) [47], the daily exposure time (*ET*), exposure frequency (*EF*), and exposure duration (*ED*) for residents are approximately 3.7 h day^−1^, 365 days year^−1^, and 74.8 years, respectively. *AT_nca_* and *AT_ca_* indicate average times under exposure to noncarcinogenic and carcinogenic risks, and both are estimated at an identical value of 74.8 years. *IUR_i_* represents the inhalation unit risk of VOC species for carcinogenic risk assessment. The values of *IUR* and reference concentration (*RfC*) of VOC species are referenced from the USEPA [30]. These input parameters can assist the evaluation of potential health risks for surrounding residents in the industrial areas.

## 3. Results and Discussion

### 3.1. Overview of Temporal Variations in Concentration of Gaseous Pollutants

**Figure 2:**
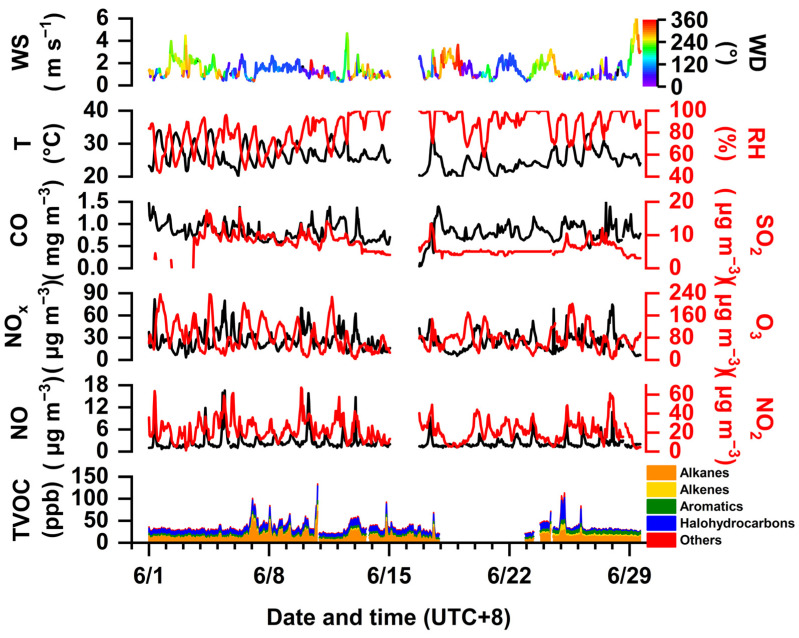
Time series of variations in concentrations of atmospheric gaseous pollutants and meteorological parameters during the field observation.

**Figure 3:**
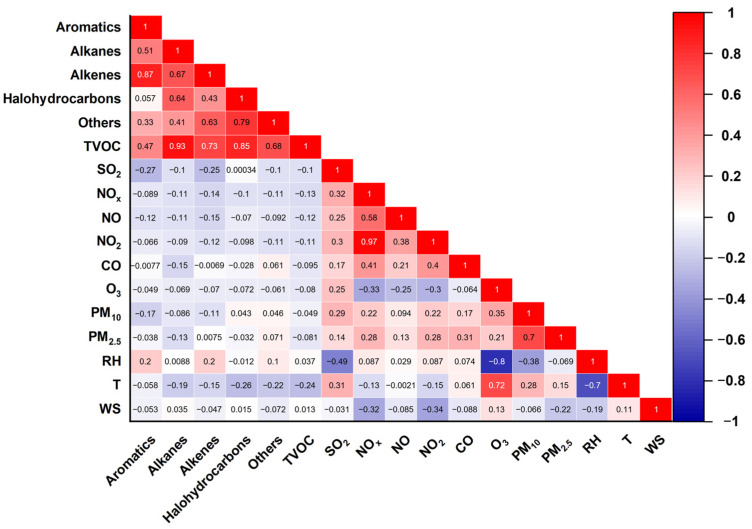
Pearson correlation analysis (*r*) of VOCs with additional trace gases and meteorological parameters.

On average, the TVOC concentrations were 34.47 ± 16.08 ppb during this summer campaign, as shown in Figure 2. Approximately 41.8% of the contribution was attributed to alkanes with 14.41 ± 8.25 ppb, followed by 29.4% attributed to halohydrocarbons (10.14 ± 5.52 ppb), 17.4% to aromatics (6.00 ± 1.58 ppb), and only 5.0% to alkenes (1.73 ± 2.58 ppb), respectively. The average temperature of 26 °C was associated with a wind speed of 1.43 m s^−1^; thereby, the meteorological conditions were characterized by high temperatures and low wind speed, which favor local accumulation of air pollutants. Situated close to the Yangtze River (see Figure 1), ambient RH was approximated to 83%, whose peak values were opposite the concentrations of trace gases such as O_3_, NO_x_, and VOCs. As shown in Figure S1, the top 10 VOC species were almost totally related to petrochemical industrial activity and traffic-related sources, such as vinyl chloride (5.67 ± 4.28 ppb) and *n*-octane (2.17 ± 1.22 ppb), respectively. The four largest long-chain alkanes above C_8_ (i.e., *n*-octane, *n*-nonane, *n*-undecane, and *n*-dodecane) were attributed most often to exhaust emissions from diesel engines [48,49]. In addition, a nonlinear response of O_3_ (79.86 ± 47.24 µg m^−3^) to NO_x_ (26.04 ± 13.03 µg m^−3^) and VOCs was exhibited throughout the observation. Peak O_3_ was present when the TVOC concentrations were constrained to a flat level between 1–7 and 27–30 June because of possible regional transport under high wind speed. VOCs showed better temporal variations with NO_x_ as compared to those between VOCs and O_3_. These signify that local industrial–vehicular sources dominated the ambient emissions, while pathways of O_3_ pollution were rather complex. Figure 3 also validates that negative correlations of TVOCs with O_3_ and NO_x_ were found at −0.33 and −0.08, respectively. In addition, different categories of VOCs showed a positive correlation with ~0.87. These observational results are in a good agreement with the pollution characteristics for complex industrial–vehicular emissions.

Many other cities beyond metropolitan areas also undergo serious VOC pollution problems, as shown in Table 1. Ambient VOCs at industrial sites exhibit higher concentrations than at urban and suburban sites owing to more intense emissions from industrial activities. Two previous field observations in industrial areas in Nanjing in 2013 and 2018 showed a high tendency similar to our observational results. TVOCs leveled off at approximately 35 ppb for the three case studies. Alkanes were the largest components and accounted for more than 40%. Conversely, aromatics have decreased since 2013 and remained at approximately 6 ppb, while alkenes have continued to decline. The VOC concentrations in highly industrialized areas in Houston were three times higher than our observational results because of large anthropogenic sources such as industrial activities, volatile chemical products (VCP), fuel evaporation, and biogenic emissions [17,50]. In addition, the growing VOC episodes are driven by complex emissions, multipollutant and meteorological conditions, and chemical processes in megapolis centers such as Shanghai and Wuhan, with ~32 ppb of TVOCs. It can be difficult to reduce air pollution in the near term for a complex urban area. By contrast, suburban or remote areas do not suffer from elevated VOC pollution. Therefore, the profit and loss associated with the strategies of emigration of non-essential industrial sectors to suburban or rural areas should be checked carefully.

### 3.2. Diurnal Patterns of Variations in VOC Emissions

**Figure 4:**
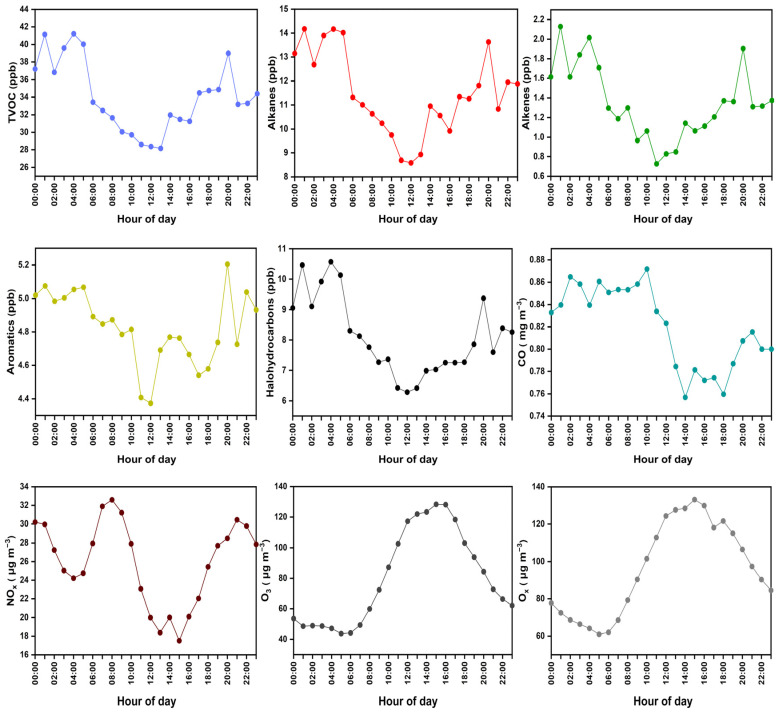
Diurnal variations in concentration of different categories of VOC groups, including alkanes, alkenes, aromatics, and halohydrocarbons, and additional trace gaseous pollutants.

The diurnal variations of alkanes, alkenes, aromatics, and halohydrocarbons exhibited a similar bimodal pattern, as shown in Figure 4. The nocturnal atmospheric environment became a considerable reservoir of primary pollutants in the urban industrial area. Considering that the oxidation capacity was reduced after sunset, concentrations of VOCs peaked at around 4 a.m. and 8 p.m., respectively, which could be ascribed to the industrial-related emissions over a typical day. According to the local traffic restriction policy, diesel vehicles can only run at night, contributing significantly to nocturnal pollution. Tracers of vehicular emissions such as n-undecane and n-octane exhibited a growing level during nighttime (Figure S2). Different from the findings in the literature, the first peak of TVOCs was advanced by 3–4 h [56,57], during which the surrounding construction sites started daily activities, accounting for additional vehicular emissions. These observations are consistent with the NO_x_ increase starting at 4 a.m. before dawn [58,59]. While CO maintained higher levels during the night, the diurnal variations of CO and NO_x_ presented a similar tendency as VOCs because they were, in part, sourced from vehicle exhaust [60]. During the daytime, high temperatures and low RH are usually associated with strong solar irradiation, which can cause build-up of O_3_ concentrations via photochemical reactions initiated by both VOCs and NO_x_ [61]. O_3_ was elevated to the maximum at noon, while VOCs and NO_x_ precursors began to decrease. Thus, a low atmospheric boundary layer linked to a relatively low wind speed and RH could help in enhancing VOCs and NO_x_ at night.

### 3.3. Pollution Characteristics of VOCs in Different Ozone Pollution Episodes

Concentrations of O_3_ tend to rise for two reasons: (1) Strong photochemical reactions can build up O_3_ formation in summer, and (2) regional transport can enhance O_3_ concentrations in downwind areas [62,63]. Table 2 lists the concentration of VOCs groups and trace gases (O_3_, CO, and NO_x_) at different O_3_ levels. O_3_ pollution often occurs at high ambient temperature and low humidity and wind speed [64]. No large variations in concentrations of VOCs were found in different ozone pollution episodes. TVOCs exhibited a slightly higher level on non-O_3_-polluted days, with 33.53 ± 17.86 ppb on average, and decreased by 1.8% to 32.94 ± 16.21 ppb on O_3_-polluted days. A similar tendency was observed for alkanes, alkenes, and aromatics [65]. However, halohydrocarbons stood out within the major categories of VOCs on O_3_-polluted days. Aside from primary anthropogenic emissions, halohydrocarbons such as halogenated compounds have been measured in the complicated reactions of chlorine radicals with O_3_ [66]. Therefore, the nonlinear response of O_3_ to reduction in VOCs in the atmosphere can be observed.

**Figure 5:**
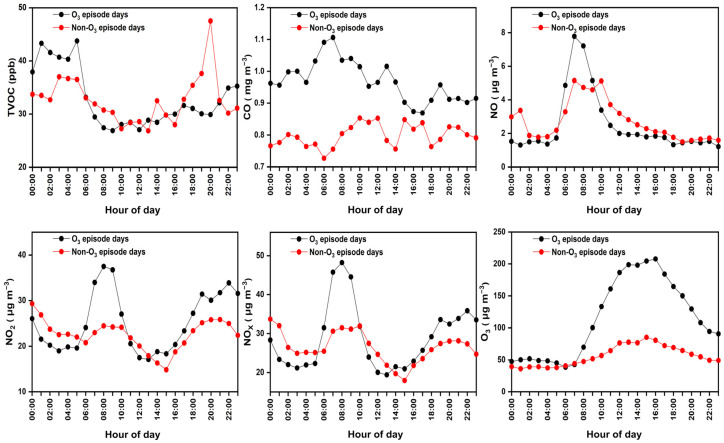
Average diurnal variations in concentration of TVOCs and additional trace gases during different ozone pollution episodes.

Trace gases increased on O_3_-polluted days, as shown in Figure 5. VOCs can react with OH to generate hydrogen peroxy radical (HO_2_) and organic peroxy radicals (RO_2_) in atmospheric chemistry [4]. In addition, they can rapidly react with NO in the atmosphere to produce NO_2_ under strong light irradiation; then, NO_2_ is consumed for O_3_ formation cycling. CO is also considered one of the important precursors of O_3_ since its reaction mechanism corresponds to the concentration of NO_x_. CO in part reacts with OH to generate HO_2_ radicals [67]. There is no huge difference in the diurnal variations of NO at different O_3_ periods (Figure 5); almost all peaks were found at 8 a.m. At this point, the concentration of CO under an O_3_-episode day was significantly higher than CO on non-O_3_-episode days, which may explain the upward concentration of O_3_ along with increased NO_x_ in the morning on O_3_-polluted days. During early nighttime, NO can be oxidized by O_3_ to cause more abundant NO_2_ on O_3_-polluted days. Concentrations of O_3_ dropped faster during O_3_-episode days and achieved around midnight a level similar to that of the clean days. However, nighttime VOCs were accumulated at a comparatively high level, possibly because the decrease in VOCs due to nocturnal ozonolysis was restricted on non-O_3_-polluted days [3]. In the meantime, intense local anthropogenic emissions contributed to VOCs accumulation over nighttime.

### 3.4. Formation Potential of O_3_ and SOA

**Figure 6:**
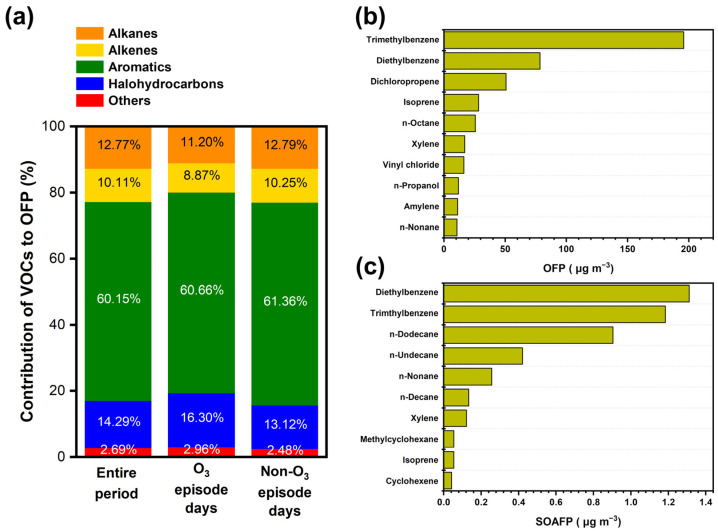
Variations in the contribution of major categories of VOCs to OFP (**a**) at different O_3_ levels. Comparisons in the top ten speciated VOCs contributing to OFP (**b**) and SOAFP (**c**).

Figure 6a,b demonstrate the fraction of TVOCs and OFP contribution from the different categories of VOC species at different O_3_ levels. Previous studies showed that regional O_3_ concentration changes are closely related to VOC emissions, and the level of VOCs plays an important role in the photochemical process of O_3_ [68,69]. MIR analysis was applied to study the contribution of VOCs species to O_3_ formation. By comparing the OFP between O_3_-episode days and non-O_3_-episode days, as shown in Figure 6b, it can be seen that halohydrocarbons decreased from 13.5% to 10.9%, while alkanes and alkenes increased from 12.3% to 13.9% and from 10.6% to 11.7%, respectively. The speciated VOCs contributing to relatively high OFP and SOAFP are compared in Figure 6c,d. The top ten speciated VOCs contributed 89.9% to the total OFP, with the sum concentration of approximately 64.5%. Aromatics accounted for the largest OFP at 61%, followed by halohydrocarbons (14%), alkanes (13%), and alkenes (10%). These results are consistent with the study that disclosed aromatics and alkenes as contributing 69.5% to the OFP [16]. These species could have high chemical reactivities with a large MIR and participate in photochemical reactions in the atmosphere. Although trimethylbenzene, diethylbenzene, and dichloropropene contributed ~8.8% to the TVOCs, they exhibited comparatively high potential for O_3_ formation, i.e., up to 40.3%, during the whole observational period and maintained similar contributions on both polluted days and non-O_3_-polluted days. Quantitative analysis of SOA formation has remained uncertain or underestimated in chemically complex environments because it cannot be ruled out by the currently accepted chemical mechanisms. The total SOAFP leveled off 4.54 µg m^−3^, of which aromatics, alkanes, and alkenes contributed 2.65 µg m^−3^, 1.80 µg m^−3^, and 0.09 µg m^−3^, respectively, with ~58.3% of SOAFP. To be specific, the top 10 speciated VOCs species contributing to SOAFP were diethylbenzene (1.31 µg m^−3^), trimethylbenzene (1.19 µg m^−3^), *n*-dodecane (0.91 µg m^−3^), *n*-undecane (0.43 µg m^−3^), *n*-nonane (0.25 µg m^−3^), *n*-decane (0.13 µg m^−3^), xylene (0.12 µg m^−3^), methylcyclohexane (0.05 µg m^−3^), isoprene (0.05 µg m^−3^), and toluene (0.04 µg m^−3^). Past study has shown similar observational results in Beijing, during which the total SOAFP was 7.9 µg m^−3^, and the two largest contributing species were aromatics and alkanes with 86.1% [37]. Although values as low as 0.9 µg m^−3^ of SOAFP were found in suburban Nanjing in 2018, aromatics made a significant contribution as well [56]. Aromatics were found to play a dominant role in in the formation of SOA, even though their concentration was only 17.4% of the TVOCs. Therefore, mitigating the emission of aromatics would help in the reduction of SOA formation.

### 3.5. Source Appointment

**Figure 7:**
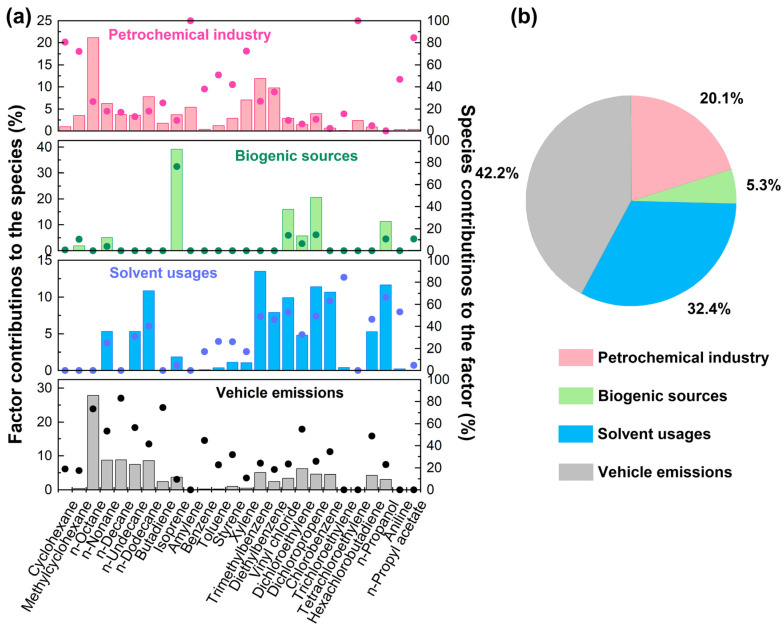
Source profiles of the four PMF-resolved factors (solid dots signify the factor contributions to each species, while bars are related to the contributions of each species to the factors) (**a**). Relative contributions of the PMF-resolved sources to TVOCs (**b**).

Quantitative determinations of dominant sources contributing to VOC emissions were investigated though the PMF model. Four factors including petrochemical industry, biogenic source, solvent usage, and vehicle emissions were resolved, as shown in Figure 7. Factor 1 was identified as the petrochemical industry, characterized by long-chain alkanes (>C_5_) and several aromatics such as cycloalkanes, amylene, and aromatic hydrocarbons. Among these species, cyclohexane and methylcyclohexane were associated with petrochemical industrial processes and fuel evaporation, accounting for 80.5% and 72.1%, respectively [49]. Additional benzene homologues containing benzene, toluene, and xylene were majorly derived from the petrochemical industry [70]. Factor 2 was characterized by a proportion of isoprene as high as 76.4%, which is a known tracer for biogenic emissions [71]. Considerable emissions from the mountain and farming areas to the south and northeast of the sampling site could have contributed to the biogenic source. Factor 3 was assigned to solvent usage and characterized by a high proportion of halogenated hydrocarbons and trimethylbenzene. Vinyl chloride, *n*-propanol, chlorobenzene, and trichloroethylene are important volatile chemical products (VCP) produced in the solvent usage associated industrial processes [72,73]. At this point, VCP emissions should be paid more attention because VCP is generated from enhanced industrial activities. Trimethylbenzene has been extensively used in solvent utilization, which is related to the solvent usage source with a contribution of 49.1%. Factor 4 was correlated with a high proportion of straight-chain alkanes (~C_8_), containing *n*-octane, *n*-nonane, *n*-decane, *n*-undecane, and *n*-dodecane, with a high contribution of 41.6–83.2%. Butadiene is an important tracer for vehicle emissions, with a 74.7% contribution. [74,75]. Factor 4 was thus assigned as a vehicle source. Based on the results of the source apportionment, the contributions of the petrochemical industry, biogenic sources, solvent use, and vehicle emissions to TVOCs are 20.1%, 5.3%, 32.4%, and 42.2%, respectively. Traffic and industrial emissions were dominant sources of ambient VOCs.

### 3.6. Health Risk Assessment

**Figure 8:**
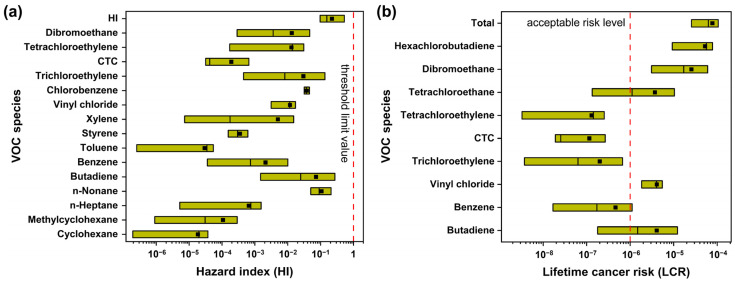
Assessments of hazard index (HI) (**a**) and lifetime cancer risk index (LCR) (**b**) for residents’ noncarcinogenic and carcinogenic risk via inhalation exposure to VOCs. The box plot represents the 5th–95th percentiles of HI and LCR. The middle line and middle square are the median values and mean values, respectively.

Many studies have shown the noncarcinogenic and carcinogenic risks of VOCs, which contain toxic and odorous components for workers [30,76]. A daily exposure time (ET) of 8 h day^−1^ has been widely used to assess occupational health risks. However, the risk assessment for surrounding residents is less-often considered due to the inability to accurately estimate exposure time and exposure frequency for residents. Here, the ET was estimated using an average exposure time for residents of 3.7 h day^−1^ when outdoors, according to the Chinese Exposure Factors Handbook (Adults) [47]. Figure 8a,b assess non-carcinogenic and carcinogenic risks for residents potentially exposed to ambient VOCs according to the industrial area via hazard index (HI) and lifetime cancer risk (LCR), respectively. The total HI value was 0.22 ± 0.24, with a median of 0.18, which is smaller than the threshold limit of 1, indicating that TVOCs did not exceed the threshold of non-cancer risks. The *n*-nonane (0.11 ± 0.06) and 1,3-butadiene (0.07 ± 0.15) accounted for 81.8% of the total HI. In addition, the average LCR value of TVOCs was 7.77 ± 8.69 × 10^−5^ higher than the acceptable risk level (10^−6^), signifying a potential carcinogenic risk to surrounding residents. The top five LCR species exceeding the acceptable level were hexachlorobutadiene (3.96 ± 3.08 × 10^−5^), dibromoethane (2.53 ± 2.84 × 10^−5^), butadiene (4.07 ± 6.92 × 10^−6^), vinyl chloride (4.09 ± 2.29 × 10^−6^), and tetrachloroethane (3.70 ± 6.05 × 10^−6^), the sum of which accounted for 98.8% of the total LCR. Besides reduction in O_3_ precursors, it is also a priority to control the emissions of hazardous or/and toxic compounds.

**Figure 9:**
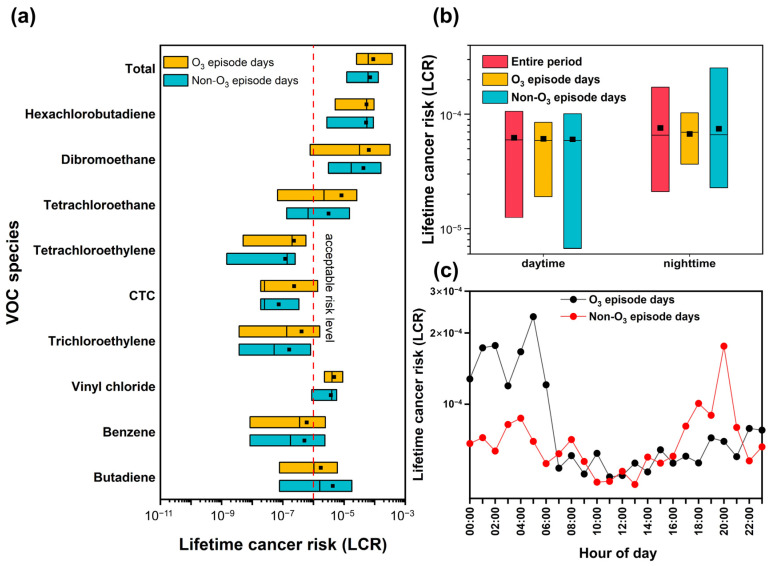
Comparisons of the LCR of priority VOC species for surrounding residents (**a**), LCR during daytime and nighttime (**b**), and diurnal variations in LCR (**c**) between O_3_-episode days and non-O_3_-episode days. The box plot represents the 5th–95th percentiles of LCR. The middle line and middle square are the median values and mean values, respectively.

As shown in Figure 9, the LCR of VOCs exhibited a relatively high level on O_3_-episode days but slightly lower concentrations of VOCs than those on non-O_3_-polluted days (see Table 2). The total LCR on O_3_-episode days was 9.0 × 10^−5^, which is higher than the 7.1 × 10^−5^ on non-O_3_-episode days (Figure 9a). Among the nine carcinogenic VOC species that were commonly found in different O_3_ episodes, a group of seven halohydrocarbons posed negligible adverse health risks and corresponded to the second-largest contribution to TVOCs in the industrial area. Figure 9b,c compare the diurnal variations of LCR (daytime was defined as 6 a.m.–6 p.m. and nighttime as 6 p.m.–6 a.m.), during which all of the LCR exceeded the acceptable risk level. In addition, unfavorable nocturnal conditions such as lower boundary layers and lower temperatures and wind speeds could hinder the diffusion of VOCs. Consequently, the LCR at nighttime was as high as 7.6 × 10^−5^ as compared to 6.24 × 10^−5^ at daytime during the entire sampling period. On O_3_-polluted days, the LCR started to increase late at night before reaching the maximum of 2.34 × 10^−4^ at around 5 a.m., then minimizing at noon, largely because the carcinogenic risks could be abated by rapid photochemical reactions of VOCs (e.g., ozonolysis). By contrast, the LCR showed a similar diurnal patten on non-O_3_-polluted days, though the peak value was exhibited at 8 p.m. with 1.76 × 10^−4^. These observations are in good agreement with the high nighttime VOCs accumulation and emissions originating from industrial-related sources, as shown in Figure 4.

## 4. Conclusions

In this study, ambient concentrations of VOCs in industrial urban areas were measured, and the associated carcinogenic risks were systematically assessed in a summer field campaign, during which strong solar irradiation can incur O_3_ pollution in a typical year. On average, concentrations of TVOCs leveled off at 34.47 ± 16.08 ppb, with alkanes and aromatics as the dominant contributing species, which is consistent with the pollution characteristics of complex industrial–vehicular emissions. Moreover, VOCs showed better temporal variations with NO_x_ (−0.08) compared to those between VOCs and O_3_ (−0.33). The diurnal variation of TVOCs exhibited a bimodal pattern that presented at night. The initial TVOC peak was observed at 4 p.m. owing to industrial activities and the civil construction-related traffic rush before dawn. In particular, diesel vehicles can only run on the road at night, which contributed to nocturnal emissions significantly. These findings were supported by the presence of the largest ~C_8_ alkanes, such as *n*-octane and *n*-nonane, which are in part related to diesel vehicle emissions. In addition, a low atmospheric boundary layer linked to low wind speed and RH assisted in enhancing nighttime VOCs and NO_x_. Aromatics played a leading role in secondary pollution, as they contributed 61% of the OFP, although their concentrations accounted for 16% of TVOC. Additional prominent contributions to SOAFP were also made by aromatics on different O_3_-polluted days, during which diethylbenzene, trimethylbenzene, and *n*-dodecane were the top three contributors to SOA formation. Aside from petrochemical industrial processes, vehicle emissions linked to on-road traffic and non-road machinery operation elevated ambient VOCs. PMF source analysis identified four sources of VOCs, namely the petrochemical industry, biogenic sources, solvent usage, and vehicle emissions. Among them, the contribution of vehicle emissions was more than 40%, while the petrochemical industry comprised 32.4%. Abating VOC emissions from vehicles could be an effective approach to controlling O_3_ pollution. In addition, the HI and LCR of VOCs were studied to evaluate the health risks for surrounding residents. While the non-cancer risk via HI of approximately 0.18 did not cross the acceptable threshold, more toxic species such as butadiene, vinyl chloride, and dibromoethane posed carcinogenic risks. Corresponding with the increased VOC concentrations, the carcinogenic risk was elevated at nighttime on O_3_-polluted days and was up to 2.34 × 10^−4^ higher than the recommended threshold of 10^−6^. It should be noted that removal of carcinogenic toxins from air is a high priority for future air-pollution control. In the context of complex air pollution in urban industrial areas, future control of alkanes and aromatics co-emitted from industrial and traffic emissions will not only reduce secondary pollution but also inhibit the acute health risks of toxic species for humans.

## Figures and Tables

**Table 1:** Comparisons of TVOC emissions with concentrations (ppb) and proportion (%) in different cities.

Sampling Sites	Duration	Alkanes ppb (%)	Aromatics ppb (%)	Alkenes ppb (%)	Halohydrocarbons ppb (%)	TVOC (ppb)	References
Shanghai, China (urban area)	2007–2010	13.91 (43.0)	9.70 (30.0)	1.94 (6.0)	4.53 (14.0)	32.35	Cai et al., 2010 [51]
Wuhan, China (urban area)	26 April–6 June, 2017	14.79 (51.1)	2.25 (7.8)	2.90 (10.0)	3.16 (10.9)	28.92	Hui et al., 2020 [52]
Taiwan, China (suburban area)	March 2012	2.28 (8.4)	7.55 (27.8)	–	0.67 (2.5)	27.17	Cheng et al., 2016 [53]
Nagoya, Japan (suburban areas)	December 2013–November 2014	16.43 (26.8)	5.58 (19.3)	4.93 (17.0)	–	28.93	Saito et al., 2009 [54]
Houston, U.S. (industrial area)	September 2006	83.69 (82.0)	22.13 (21.7)	16.99 (16.6)	0.09 (0.1)	102.1	Leuchner et al., 2010 [50]
Aliaga, Turkey (industrial area)	July 2009–April 2010	15.96 (65.8)	4.00 (16.5)	3.06 (12.6)	1.15 (4.7)	24.24	Dumanoglu et al., 2014 [55]
Nanjing, China (industrial area)	15 May–31 August 2013	14.98 (43.5)	9.06 (26.3)	7.35 (21.4)	–	34.40	Shao et al., 2016 [41]
Nanjing, China (industrial area)	3 June–1 August 2018	14.35 (41.0)	5.60 (16.0)	3.15 (9.0)	–	35.00	Mozaffar et al., 2020 [56]
Nanjing, China (industrial area)	1–30 June 2020	14.41 (41.8)	6.00 (17.4)	1.73 (5.0)	10.14 (29.4)	34.47	This study

**Table 2:** Comparisons in concentrations of VOCs with additional trace gases and variations in fraction (%) of major categories of VOCs to the TVOCs in different ozone pollution episodes.

Parameters and Gases	Entire Period	O_3_-Polluted Days	Non-O_3_-Polluted Days
T (°C)	26	28	25
WS (m s^−1^)	1.4	1.1	1.5
RH (%)	83	72	93
CO (mg m^−3^)	0.82 ± 0.20	0.97 ± 0.17	0.80 ± 0.22
NO (µg m^−3^)	2.49 ± 1.92	2.49 ± 2.06	2.74 ± 2.34
NO_2_ (µg m^−3^)	22.33 ± 11.45	25.31 ± 9.60	22.65 ± 13.03
NO_x_ (µg m^−3^)	26.04 ± 13.03	28.92 ± 11.55	26.64 ± 12.30
TVOCs (ppb)	34.47 ± 16.08	32.94 ± 16.21	33.53 ± 17.86
Alkanes ppb (%)	14.41 ± 8.25 (41.80)	12.36 ± 5.21 (37.52)	14.29 ± 9.66 (42.62)
Alkenes ppb (%)	1.73 ± 2.58 (5.02)	1.55 ± 2.67 (4.71)	1.74 ± 2.95 (5.19)
Aromatics ppb (%)	6.00 ± 1.58 (17.41)	5.83 ± 1.67 (17.70)	6.13 ± 1.67 (18.28)
Halohydrocarbons ppb (%)	10.14 ± 5.52 (29.42)	10.75 ± 6.85 (32.64)	9.40 ± 5.50 (28.03)

## Data Availability

The data presented in this article are available on request from the corresponding author.

## Supplementary Materials

The following supporting information can be downloaded at: https://www.mdpi.com/article/10.3390/toxics12010034/s1, Figure S1: List of top ten of concentrations of VOC species; Figure S2: Diurnal variations in the concentration of n-octane and n-undecane tracers for diesel engine emissions; Table S1: Major identified compounds from mass list of SPI-ToF-MS.
