# Supplementary material for: Significance of Volatile Organic Compounds to Secondary Pollution Formation and Health Risks Observed during a Summer Campaign in an Industrial Urban Area
Source: Toxics. 2024 Jan 1;12(1):34. doi: 10.3390/toxics12010034 (PMC10820161; doi:10.3390/toxics12010034)
Supplement: Supplementary file 1 [file toxics-12-00034-s001.zip › toxics-2768261-supplementary.pdf]

Supplementary Materials for

Significance of Volatile Organic Compounds to Secondary Pollution Formation and Health Risks Observed during a Summer Campaign in an Industrial Urban Area

Li Cao [1], Qihui Men [1], Zihao Zhang [1], Hao Yue [1], Shijie Cui [1], Xiangpeng Huang [2], Yunjiang Zhang [1], Junfeng Wang [1], Mindong Chen [1] and Haiwei Li [1,3,*]

[1] Jiangsu Key Laboratory of Atmospheric Environment Monitoring and Pollution Control, Collaborative Innovation Center of Atmospheric Environment and Equipment Technology, School of Environmental Science and Engineering, Nanjing University of Information Science and Technology, Nanjing 210044, China

[2] Shanghai Key Laboratory of Atmospheric Particle Pollution and Prevention, Department of Environmental Science and Engineering, Fudan University, Shanghai 200438, China

[3] State Environmental Protection Key Laboratory of Formation and Prevention of Urban Air Pollution Complex, Shanghai Academy of Environment Sciences, Shanghai 200233, China

* Correspondence: haiwei.li@nuist.edu.cn

Supplementary Information: Figure & Table Captions

Figure S1. List of top ten of concentrations of VOC species.

Figure S2. Diurnal variations in the concentration of *n*-octane and *n*-undecane tracers for diesel engine emissions.

Table S1. Major identified compounds from mass list of SPI-ToF-MS.

Supplementary text 1: Calibrations of SPI-ToF-MS

The real-time VOC speciation and concentrations were measured using single photon ionization time-of-flight mass spectrometer (SPI-ToF-MS 3000, Guangzhou Hexin Instrument Co., Ltd., Guangzhou, China). The SPI-MS is composed of sampling inlet system, ion source and time-of-flight (ToF) mass analyzer for high time-resolution monitoring of VOCs with a 1-minitute interval. The sampling inlet system is equipped with Polydimethylsiloxane (PDMS) membrane for speciated VOC analyte. The PDMS membrane can filter the interfering inorganic substances because the diffusion rate of VOCs is higher than that of inorganic gas before entering into the ion source. The ion source of the SPI-MS mainly consists of vacuum ultraviolet lamp (VUV), ionization chamber, and electrode-lens. The VUV lamp, mounted on the left side of ionization chamber, is a krypton discharge lamp with ionization energy of 10.8 eV. The speciated VOCs with ionization energy no more than 10.8 eV can be ionized by VUV photoionzation. The mass spectrometer consists of accelerate region, drift region, reflector region, and micro channel plate detector. Standard benzene and toluene mixture gases carried by ultrapure N_2 feed gas (99.999%) were used to examine routine measurement. Diffent concentrations of benzene and toluene in the experiments were obtained through a dynamic gas dilution instrument (Sabio 2010, SABIO company, Round Rock, Texas, USA). Prior to field observations, the resolution and linear range of different components of standard gas mixture were obtained through the test of standard gas mixture. Major identified compounds from mass list of SPI-ToF-MS are given in **Table S1**, the regression equation and limit of detection VOCs were obtained via the calibration using PAMS and TO-15 standard gases. Standard gases were diluted to concentrations of 0, 1, 2, 5, 10, 20, and 40

ppb for calibration. The flowrate of sampling was 1 L min^{-1}. The correlation coefficient (R^2) of the calibration curves was ~0.99 for different 46 VOCs, and each calibration was maintained at least 5 times through standard gases until to reach equilibrium.

Supplementary Figures

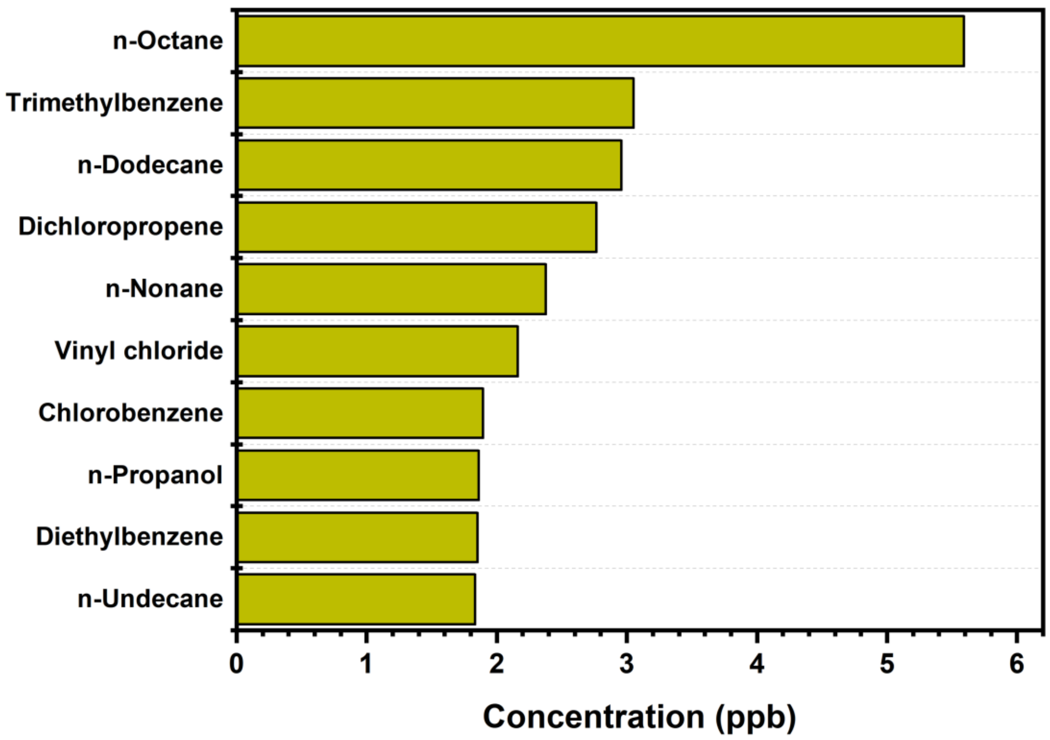

Figure S1. List of top ten of concentrations of VOC species.

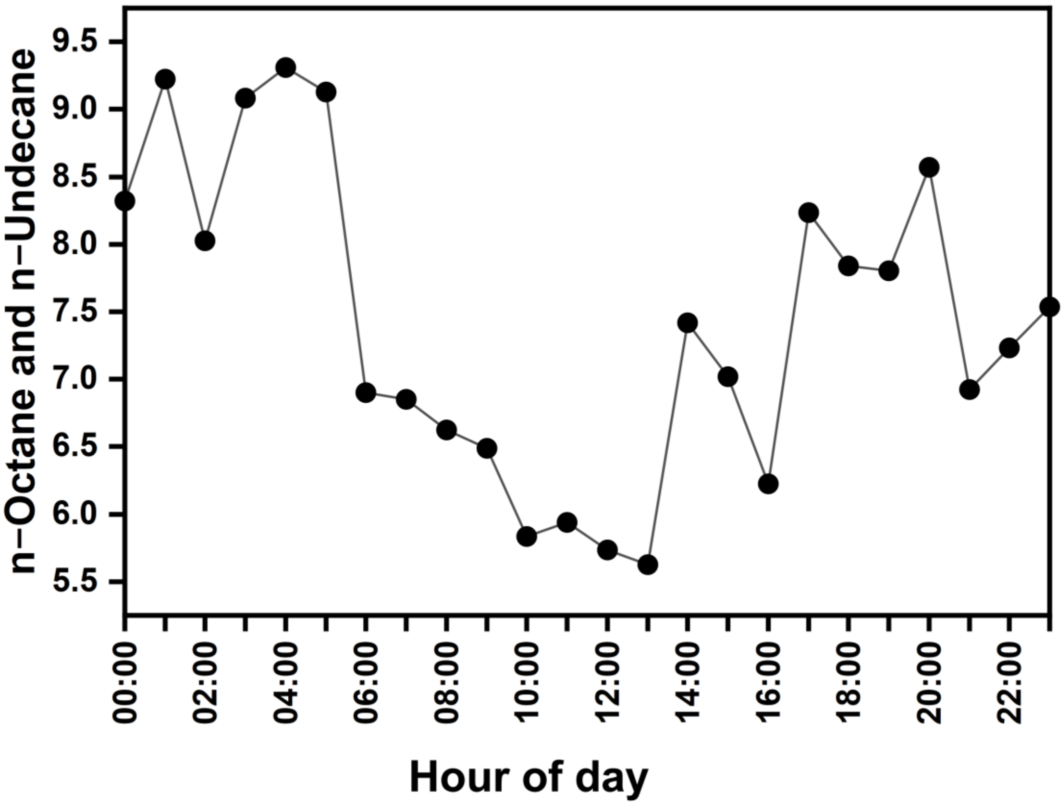

Figure S2. Diurnal variations in the concentration of *n*-octane and *n*-undecane tracers for diesel engine emissions.

Supplementary Table

Table S1. Major identified compounds from mass list of SPI-ToF-MS.

**Speciated VOCs**		**LOD (ppb)**	**Signal sensitivity (R^2)[1]**	**Speciated VOCs**		**LOD (ppb)**	**Signal sensitivity (R^2)[1]**
	Butane	0.5	0.99		Dichloroethylene	0.66	0.99
	Pentane	0.5	0.99		Dichloropropene	0.37	0.99
	Cyclohexane	0.5	0.99		Chlorobenzene	0.09	0.99
	Methylcyclohexane	1.49	0.99		Trichloroethylene	0.04	0.99
	n-Heptane	1.49	0.99	halohydrocarbon	Carbon tetrachloride	0.5	0.99
alkane	n-Octane	0.36	0.99		Tetrachloroethylene	0.47	0.99
	n-Nonane	0.89	0.99		Tetrachloroethane	1.7	0.99
	n-Decane	1.7	0.99		Dibromomethane	0.5	0.99
	n-Undecane	1.61	0.99		Dibromoethane	0.62	0.99
	n-Dodecane	1.37	0.99		Hexachlorobutadiene	0.04	0.99
	n-Tridecane	0.5	0.99		n-Propanol	0.5	0.99
	Butadiene	0.49	0.99		Ethyl formate	0.5	0.99
	Butene	4.02	0.99	oxygenated compound	n-Propyl acetate	0.5	0.99
alkene	Isoprene	0.79	0.99		n-Butyl acetate	0.5	0.99
	Amylene	3.22	0.99		Xylenol	0.5	0.99
	Cyclohexene	0.5	0.99		Methyl benzoate	0.5	0.99

aromatic	Benzene	0.06	0.99	nitrogen-containing compound	Trimethyl phosphate	0.5	0.99
	Toluene	0.16	0.99		n-Decanol	0.5	0.99
	Styrene	0.02	0.99		Dimethylformamide	0.5	0.99
	Xylene	0.41	0.99		Aniline	0.5	0.99
	Trimethylbenzene	1.31	0.99		Triethylamine	0.5	0.99
	Diethylbenzene	0.12	0.99		Diethylenetriamine	0.5	0.99
halohydrocarbon	Vinyl chloride	0.5	0.99		Diethyl aniline	0.5	0.99

[1] The correlation coefficient (R^2) of the calibration curves for speciated VOCs.
